# Serum lactate and capillary refill time as predictors of acute kidney injury after pediatric cardiovascular surgery: a prospective cohort study

**DOI:** 10.1007/s00467-026-07303-9

**Published:** 2026-07-23

**Authors:** Vanesa A. Ochoa Jimenez, Inés E. Gómez Hernández, Ana María Portilla Buenaventura, Alejandro Padilla Guzman, Camilo José Cuadros Mateus, Christian Pinilla-Medina, Gustavo Adolfo Cruz Suarez, Carlos Alberto González Amador, Juan Fernando Vélez Moreno, María del Pilar Duque Guevara, Jesús Aldemar Aguirre Recalde, Laura Torres Canchala, Jessica M. Forero Delgadillo, Jaime Manuel Restrepo

**Affiliations:** 1 Department of Pediatrics, Pediatric Nephrology Service, Fundación Valle del Lili, Cali, Colombia; 2 Facultad de Ciencias de La Salud, Universidad Icesi, Cali, Colombia; 3 Centro de Investigaciones Clínicas, Fundación Valle del Lili, Cali, Colombia; 4 Department of Anesthesiology, Fundación Valle del Lili, Cali, Colombia; 5 Department of Pediatrics, Universidad del Valle, Cali, Colombia; 6 Pediatric Cardiovascular Intensive Care Unit, Fundación Valle del Lili, Cali, Colombia

**Keywords:** Acute kidney injury, Congenital heart surgery, Serum lactate, Capillary refill time, Pediatric critical care, RACHS1

## Abstract

**Background:**

Acute kidney injury (AKI) is a frequent complication after pediatric cardiovascular surgery (CS) and is associated with higher morbidity and mortality. Early, modifiable predictors are needed to improve outcomes.

**Methods:**

We conducted a prospective cohort study at a high-complexity center in Cali, Colombia (November 2020–July 2022). Children aged 0–60 months undergoing CS and admitted to the pediatric cardiovascular ICU were included. AKI was defined and staged according to KDIGO criteria. Serum lactate and capillary refill time (CRT) were measured preoperatively, at ICU admission, and at 6, 12, and 24 h postoperatively. Univariate and multivariate Cox proportional hazards models were used to assess associations with AKI.

**Results:**

Of 127 patients, 14.17% developed AKI, 66.7% of which were severe. The 28-day mortality was 11.8% overall and 44.4% among patients with AKI. In the multivariate analysis, elevated serum lactate was associated with higher risk of AKI at 24 h postoperatively (HR 1.61; 95%CI 1.24–2.08). Prolonged CRT at ICU admission was also a significant predictor (HR 1.80; 95%CI 1.03–3.15). Higher surgical complexity (RACHS-1), vasopressor use, and low systolic blood pressure were also associated with AKI.

**Conclusions:**

In this cohort, postoperative tissue perfusion markers—elevated serum lactate and prolonged CRT—were associated with AKI following pediatric CS. These accessible bedside measures may support early risk stratification and targeted kidney protective strategies.

**Graphical abstract:**

**Figure:**
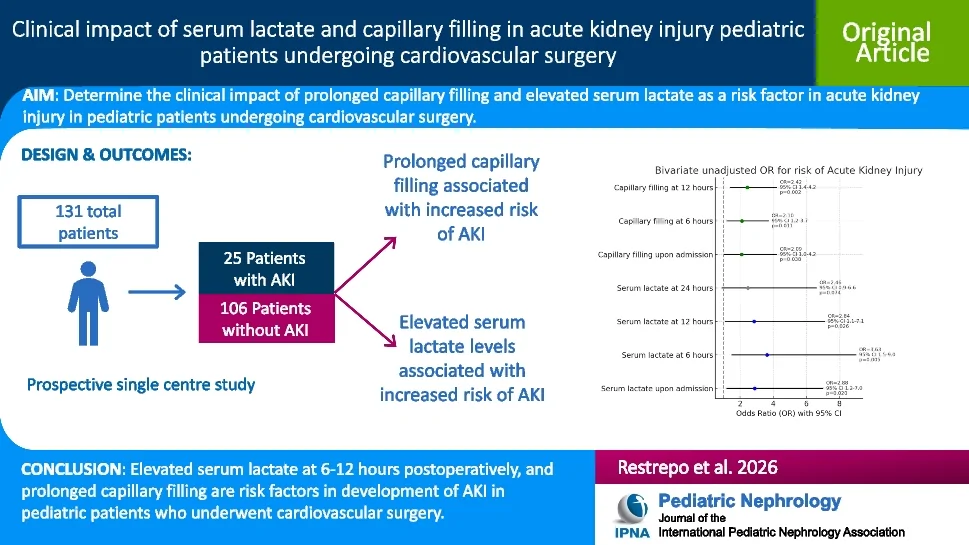
A higher resolution version of the Graphical abstract is available as Supplementary information

**Supplementary Information:**

The online version contains supplementary material available at https://doi.org/10.1007/s00467-026-07303-9.

## Introduction

Acute kidney injury (AKI) is defined as a sudden decline in renal function that may be reversible. It represents a common complication among hospitalized and critically ill patients [1–3]. In the pediatric population, AKI following cardiovascular surgery has been reported with a global incidence of 34.3% (95%CI 30.0–38.0%) [4]. In Latin America, however, data remain limited. Restrepo de Rovetto et al. reported an incidence of 18.3% [5], while Cardoso et al. described an incidence of 12.3% on the first postoperative day [6].

AKI is recognized as a strong predictor of morbidity and mortality; nonetheless, when identified at an early stage, preventive strategies can be implemented to mitigate its impact [7, 8]. The pathophysiological association between AKI and pediatric cardiovascular surgery is multifactorial, involving mechanisms such as low cardiac output, systemic release of proinflammatory cytokines, ischemia–reperfusion injury, mechanical trauma, oxidative stress, and exposure to nephrotoxic agents.

Evidence from 2000 to 2012 highlights the role of serum lactate as a marker of morbidity and mortality after cardiovascular surgery. Basaran et al. conducted a study in 60 patients undergoing cardiovascular surgery and reported that 26 patients (43.3%) had serum lactate levels greater than 4.8 mmol/L, while 34 patients (56.7%) had levels less than or equal to 4.8 mmol/L. Elevated serum lactate (≥ 4.8 mmol/L) in the early postoperative period identified a subgroup of patients at high risk of perioperative morbidity and mortality [9].

Although advances in serum and urinary biomarkers have improved the early detection of AKI, it remains essential to rely on simple, widely available diagnostic tools such as serum creatinine and serum lactate. Venous oxygen saturation and serum lactate are among the most frequently used parameters for monitoring tissue oxygen supply and consumption. Lactate, as a product of anaerobic metabolism, reflects tissue hypoperfusion and has proven to be more sensitive for detecting inadequate oxygen delivery or imbalance between oxygen supply and metabolic demand [10].

Capillary refill time (CRT) measures the amount of time necessary for the skin to return to baseline color after the application of a firm pressure [11]. CRT is a quick, cost-free method that allows tissue perfusion assessment and is available at the patient’s bedside [12, 32]. It also responds faster to change than lactate clearance after hypoperfusion is addressed [13, 33].

It has not yet been fully explored whether CRT and serum lactate are perfusion markers that can serve as predictors of AKI in critically ill pediatric patients undergoing cardiovascular surgery [14, 15]. Based on this rationale, the present study was designed to evaluate these markers as predictors of AKI in postoperative pediatric patients following cardiovascular surgery.

## Methods

### Study design and setting

A prospective cohort study was conducted at a high-complexity institution in Cali, Colombia, which included 135 patients between 0 and 60 months of age, undergoing cardiovascular surgery and admitted to the Pediatric Cardiovascular ICU between November 2020 and July 2022. Patient follow-up was recorded in electronic medical records, and subsequently the variables were collected and stored in a custodial database, which was only accessible to the research team. This study was conducted and reported in accordance with the Strengthening the Reporting of Observational Studies in Epidemiology (STROBE) guidelines for cohort studies.

### Participants

All of the children aged 0–60 months who underwent congenital cardiac surgery and were admitted postoperatively to the pediatric cardiovascular intensive care unit (ICU) were eligible. Patients with a history of chronic kidney failure or lacking outcome ascertainment were excluded.

### Variables and definitions

Clinical and paraclinical variables were obtained before and after surgery from the patients included in the study, such as, age, sex, pathological history (congenital malformations, prematurity, lung disease, kidney disease, liver disease), pharmacological history, anthropometric measurements (weight and height), exposure to nephrotoxic drugs prior to surgery (aminoglycosides, beta-lactams, diuretics, angiotensin-converting enzyme (ACE) inhibitors, ARBs), type of cardiovascular surgery, Risk Adjustment for Congenital Heart Surgery-1 (RACHS-1) scale, blood count and renal function before and after surgery (6, 12 and 24 h), requirement of inotropic drugs, vasopressors or transfusion of blood products during surgery, time on the pump, and clamping time. Preoperative creatinine readings were adopted as baseline. After surgery, information was collected on the outcomes of interest (incidence of AKI and mortality). Exposure to nephrotoxic drugs before surgery was recorded throughout the patient’s hospital stay using the drug monitoring program from the pharmacology group at the Fundación Valle del Lili Hospital.

The glomerular filtration rate was estimated using the modified Schwartz formula [16, 37] and the diagnosis of AKI was at any time within the first 8 postoperative days. The diagnostic criteria included an increase in serum creatinine of ≥ 0.3 mg/dL within 24 h, an increase in serum creatinine to 1.5 times the baseline value within the preceding 8 days, or a urinary output of ≤ 0.5 mL/kg/h for 6 h. All cases were classified according to the KDIGO scale [6].

Shock was defined as a clinical state of inadequate tissue perfusion requiring vasopressor and/or inotropic support and resulting in anaerobic metabolism, lactate accumulation, and dysfunction of vital organs. In addition, it was classified into 4 categories: hypovolemic, distributive, cardiogenic, and obstructive according to the clinical criteria reported in the scientific evidence [6, 14, 17].

Serum lactate was measured by using point of care testing through the I-STAT and capillary filling was measured prior to surgery, at admission to the ICU CV, in the immediate postoperative period, and at 6, 12, and 24 h later. Capillary filling measurements were performed by 2 people (the cardiovascular anesthesiologist and the ICU physician). Firm pressure was applied for 5 s to the patient’s right heel (if not possible, it was performed on the ventral surface of the distal right index finger) and capillary filling was measured with a stopwatch. The patient’s temperature had to be equal to or greater than 35.9 °C and the temperature in the ICU cubicle had to be greater than 22 °C. 25% of the measurements were corroborated by another observer, obtaining a 100% agreement.

### Exposures and measurements

The prespecified exposures were serum lactate (mmol/L) and CRT (seconds). Lactate and CRT were measured preoperatively, at ICU admission (immediate postoperative), and 6, 12, and 24 h after surgery. For analysis, lactate was treated as a continuous predictor per 1 mmol/L increase; CRT as a continuous variable in seconds. Units were standardized across text and tables.

### Outcomes

Primary outcomes include the incidence of AKI in patients undergoing cardiovascular surgery and determining if elevated serum lactate and prolonged CRT are associated with the development of AKI.

Secondary outcomes included stage 2–3 AKI distribution, other variables associated with AKI presentation, invasive mechanical ventilation (IMV) days, ICU and hospital length of stay, postoperative ECMO, in-hospital mortality, and 28-day mortality.

### Missing data

When constructing the univariate and multivariate models, it was necessary to censor patients who developed AKI after 8 days.

### Statistical analysis

A descriptive analysis of the data was performed according to the nature of the variables and their distribution. The incidence of AKI was calculated as the proportion of detected cases over the total number of patients admitted to the cardiovascular ICU during the study period. The population was divided between patients with and without a diagnosis of AKI, comparing the variables of interest. Continuous data were analyzed using parametric (Student’s *t*-test) or nonparametric (Wilcoxon rank sum test) methods, depending on whether normality assumptions were met. Categorical variables were compared using the Chi-square test (*χ*^2^) or Fisher’s exact test.

To assess the association between potential risk factors and AKI, an initial univariate Cox proportional hazards model was applied, analyzing each predictor separately. Variables that showed statistical significance (*p* < 0.05) in the univariate analysis were subsequently included in multivariate models to adjust for potential confounding factors. Because measurements were obtained at different follow-up points (Cardiovascular ICU admission, and at 6, 12, and 24 h), separate models were constructed for each time point, considering the longitudinal nature of the data. For each model, a diagnosis of the assumptions of the Cox multivariate proportional hazards model was performed, including: evaluation of influential observations using DFBETAs, analysis of deviance residuals to verify the functional form of the covariates, and verification of the proportional hazards assumption using the Schoenfeld test (global and individual). Additionally, the Kaplan–Meier method was used to estimate the cumulative probability of remaining free from AKI during the first 8 postoperative days. Results were expressed as hazard ratios (HR) with 95% confidence intervals. All analyses were performed using R software, version 4.5.2.

## Results

**Fig. 1:**
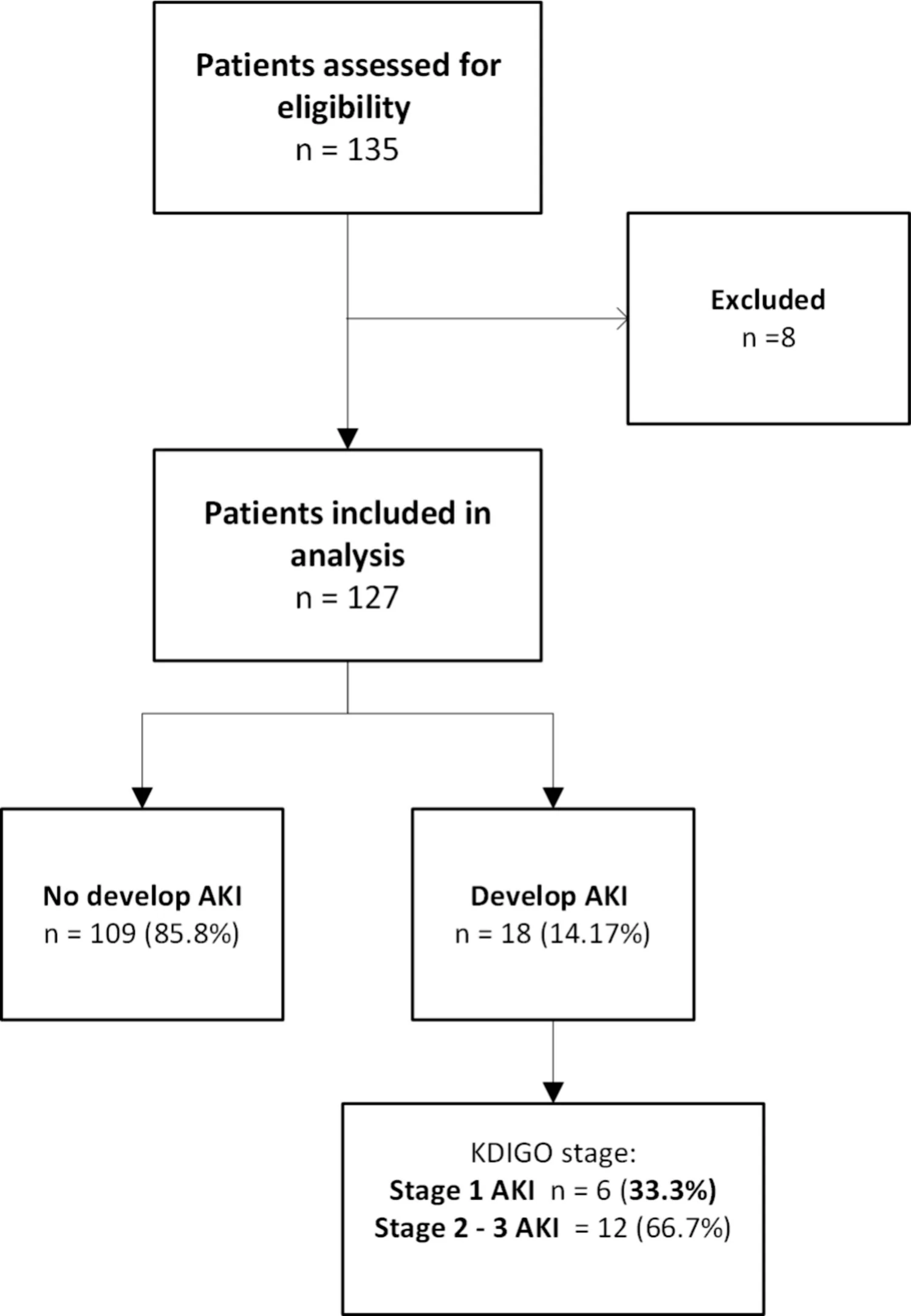
Flowchart of study participants. Of 135 patients assessed eligibility; 8 were excluded due to AKI diagnosis beyond the prespecified 8-day postoperative window. The remaining 127 patients were included in the final analysis, of whom 18 (14.2%) developed AKI; 66.7% of AKI cases were severe (KDIGO stages 2–3). AKI, acute kidney injury; KDIGO, Kidney Disease: Improving Global Outcomes

A total of 127 patients were included, of which 52.7% were male and 86.6% came from rural areas. 14.17% developed AKI. Cyanotic heart disease was the most frequent clinical history in both groups (68.8% and 66.7%, *p* = 0.9) between AKI and no AKI groups (Fig. 1). Likewise, there was no statistical difference observed in median creatinine pre-surgery (*p* < 0.8) between patients that developed AKI and those that did not.

Prior to surgery, 35 patients were exposed to various nephrotoxic drugs, being more frequent in patients who did not develop AKI (29.2%, *p* = 0.003). Among diuretics, the use of potassium-sparing agents was more frequent in patients without AKI (33% vs. 16%, *p* = 0.2). ACE inhibitors were used more frequently in patients without AKI (34.9% vs. 12%, *p* = 0.038). Regarding antibiotics, no difference in vancomycin use (11.3% vs. 16%, *p* = 0.4) or aminoglycosides (5.7% vs. 12%, *p* = 0.3) were observed in patients with AKI (see Table 1).

**Table 1:** Clinical, sociodemographic, and presurgical characteristics of pediatric patients undergoing cardiovascular surgery

Characteristics	Patients without acute kidney injury	Patients with acute kidney injury	*p*-value
	*n* = 109	*n* = 18	
Sex (*n*, %)			
Female	54 (49.5)	6 (33.3)	0.2
Male	55 (50.5)	12 (66.7)	
Age at surgery in months (median, IQR)	9 (3–48)	0 (0–2)	< 0.001
*Area of origin, n (%)*			
Urban	15 (13.8)	2 (11.1)	> 0.9
Rural	94 (86.2)	16 (88.9)	
*Clinical history, n (%)*			
Premature	13 (16.7)	3 (16.7)	> 0.9
Lung disease	26 (23.9)	6 (33.3)	0.4
Cyanotic heart disease	75 (68.8)	12 (66.7)	0.9
Chromosomopathy	30 (27.5)	3 (16.7)	0.4
CAKUT + O17:O34	5 (4.9)	-	0.836
Liver disease	3 (2.8)	1 (5.6)	0.5
*Presurgical variables, median (IQR)*			
Weight (Kg)	6.8 (4.4, 13.9)	3.5 (3.2, 4.5)	< 0.001
TFG (mL/min/m^2^)	85.5 (67.0, 107.0)	62.0 (46.0, 97.0)	0.026
Hematocrit (%)	39.3 (34.4, 45.7)	39.2 (36.0, 43.7)	> 0.9
Hemoglobin (mg/dL)	13.4 (11.5, 15.2)	13.1 (12.2, 14.7)	0.7
Creatinine (mg/dL)	0.4 (0.3, 0.5)	0.4 (0.3, 0.5)	0.8
*Exposure to nephrotoxic drugs, (n%)*			
Exposure to nephrotoxins	31 (29.2)	4 (16)	0.003
Number of nephrotoxins exposed	1 (0–4)	0 (0–2)	0.181
Potassium-sparing diuretic	34 (32.1)	4 (16)	0.085
Loop diuretic	29 (27.4)	7 (28)	0.564
Thiazide diuretic	32 (30.2)	3 (12)	0.049
ACE inhibitor	37 (34.9)	3 (12)	0.019
Angiotensin II receptor blockers	0 (0)	0 (0)	
Vancomycin	12 (11.3)	4 (16)	0.363
Aminoglycosides	6 (5.7)	3 (12)	0.233
Penicillin	5 (4.7)	2 (8)	0.399
Cephalosporin	13 (12.3)	9 (36)	0.413

Median (Q1, Q3); *n* (%)

Regarding intrasurgical clinical characteristics, no statistically significant differences were observed between the groups with and without AKI. The distribution according to the RACHS scale showed a trend towards categories of greater complexity in the group with AKI, with significantly higher percentages in levels four (13% vs. 11.1%, *p* = 0.2) and five (3.7% vs. 16.7%, *p* = 0.2), while in the group without kidney injury levels RACHS 1 and 2 predominated (48.6% vs. 28%, *p* = 0.2). There was no statistical significance between serum lactate levels, longer extracorporeal circulation times, or aortic clamp time of patients that developed AKI and patients that did not (see Table 2).

**Table 2:** Intrasurgical clinical characteristics of pediatric patients undergoing cardiovascular surgery

Characteristics	Patients without acute kidney injury	Patients with acute kidney injury	*p*-value
	*n* = 109	*n* = 18	
RACHS scale, *n* (%)			0.2
One	11 (10.3)	-	
Two	52 (48.6)	8 (44.4)	
Three	26 (24.3)	5 (27.8)	
Four	14 (13.1%)	2 (11.1)	
Five	4 (3.7)	3 (16.7)	
Serum lactate (mmol/L), median (IQR)	2.0 (1.4, 2.9)	2.5 (1.9, 5.9)	0.066
Intraoperative bypass time [min], median (IQR)	79.0 (54.0, 112.0)	137.0 (51.0, 205.5)	0.2
Clamp Time [min], median (IQR)	54.0 (30.0, 73.0)	78.0 (56.0, 127.0)	0.029

Median (Q1, Q3); *n* (%)

Regarding postoperative clinical outcomes, the PRISM score, which reflects the severity of the disease, was significantly higher in the group with kidney injury (12 vs. 7 points, *p* < 0.001). The incidence of sepsis was markedly higher in patients with kidney injury (38.9% vs. 6.4%, *p* < 0.001). Regarding the types of shock, patients with renal injury had higher rates of cardiogenic shock (61.1% vs. 17%, *p* < 0.001), hypovolemic shock (22.2% vs. 2.8%, *p* = 0.008) and distributive shock (27.8% vs. 7.3%, *p* = 0.002), with no significant differences in obstructive shock (see Table 3).

**Table 3:** Postoperative outcomes of pediatric patients with cardiovascular surgery

Features	Patients without acute kidney injury	Patients with acute kidney injury	*p*-value
	*n* = 109	*n* = 18	
PRISM score, *n* (%)	7 (4–10)	12 (9–15)	< 0.001
Sepsis, *n* (%)	7 (6.4%)	7 (38.9%)	< 0.001
Presence of shock, *n* (%)			
Cardiogenic	17 (15.6%)	11 (61.1%)	< 0.001
Hypovolemic	3 (2.8%)	4 (22.2%)	0.008
Obstructive	2 (1.8%)	0 (0)	> 0.9
Distributive	8 (7.3%)	5 (27.8%)	0.002
Cardiopulmonary resuscitation requirement, *n* (%)	4 (3.7%)	2 (11.1%)	0.2
Postoperative vasopressor use, *n* (%)	68 (62.4%)	16 (88.9%)	0.028
Postoperative ECMO requirement, *n* (%)	6 (5.5%)	7 (38.9%)	< 0.001
IMV days, median (IQR)	2 (1–6)	12 (5–36)	< 0.001
Days of hospital stay, median (IQR)	14.0 (7.0, 32.0)	39.0 (19.0, 48.0)	0.001
Days of stay in ICU, median (IQR)	7.0 (4.0, 15.0)	26.5 (14.0, 55.0)	< 0.001
Death at discharge, *n* (%)	8 (7.3%)	8 (44.4%)	< 0.001

In addition, use of vasopressors (88.9% vs. 62.4%, *p* = 0.028) and postoperative ECMO (38.9% vs. 5.5%, *p* < 0.001) were found statistically significant, with more use of both in the AKI group. The duration of invasive mechanical ventilation was significantly longer in the group with kidney injury (12 vs. 2 days, *p* < 0.001), as well as hospital stay (39 vs. 14 days, *p* < 0.001) and ICU (26.5 vs. 7 days, *p* < 0.001). Mortality at discharge was substantially higher in patients who developed AKI (44.4% vs. 7.3%, *p* < 0.001).

### Univariate model

Univariate Cox regression analysis identified several factors associated with the development of AKI in pediatric patients undergoing cardiovascular surgery, as evidenced in Table 4. It was found that the risk of AKI is associated with early tissue perfusion, with capillary filling being the most robust marker in all follow-up windows. Its maximum impact was observed 12 h postoperatively, where each additional second was associated with a significant increase in risk (HR 2.24, 95%CI 1.34–3.72, *p* = 0.002). Consistently, this clinical marker at admission to the cardiovascular ICU showed a comparable effect (HR 2.13, 95%CI 1.50–3.02, *p* < 0.001). Although the risk decreased at 24 h, it remained significant (HR 1.38, 95%CI 1.07–1.77, *p* = 0.012).

**Table 4:** Univariate Cox regression analysis

Variables		Acute kidney injury		*p*-value^*2*^
	Overall	No	Yes	
	*N* = 127^*1*^	*N* = 109^*1*^	*N* = 18^*1*^	
				< 0.001
CRT upon admission	3.0 (2.0, 4.0)	3.0 (2.0, 3.0)	3.0 (3.0, 5.0)	0.002
CRT at ICU arrival	3.0 (2.0, 4.0)	3.0 (2.0, 3.0)	3.0 (3.0, 4.0)	0.007
	1	1	0	
CRT 6 h post surgery	2.0 (2.0, 3.0)	2.0 (2.0, 3.0)	3.0 (2.0, 3.0)	0.035
	3	2	1	
CRT 12 h post surgery	2.0 (2.0, 3.0)	2.0 (2.0, 3.0)	3.0 (3.0, 3.0)	0.001
	4	4	0	
CRT 24 h post surgery	2.0 (2.0, 3.0)	2.0 (2.0, 2.0)	3.0 (2.0, 3.0)	< 0.001
	5	5	0	
Serum lactate at ICU arrival	2.0 (1.3, 3.1)	1.9 (1.2, 2.7)	4.9 (1.9, 9.9)	< 0.001
Serum lactate 6 h post surgery	1.6 (1.1, 2.5)	1.4 (1.1, 2.2)	4.1 (1.5, 7.1)	< 0.001
	13	13	0	
Serum lactate 12 h post surgery	1.3 (1.0, 2.0)	1.2 (1.0, 1.6)	2.6 (1.5, 6.4)	< 0.001
	10	9	1	
SBP at ICU arrival	97.0 (84.0, 108.0)	98.0 (89.0, 108.0)	76.0 (67.0, 92.0)	< 0.001
DBP at ICU arrival	56.0 (46.0, 64.0)	55.0 (47.0, 63.0)	58.0 (43.0, 65.0)	0.8
MBP at ICU arrival	68.0 (59.0, 78.0)	70.0 (60.0, 79.0)	64.0 (53.0, 70.0)	0.067
SBP 6 h post surgery	92.0 (75.0, 104.0)	94.0 (80.0, 106.0)	69.0 (60.0, 82.0)	< 0.001
DBP 6 h post surgery	53.0 (43.0, 62.0)	53.0 (45.0, 62.0)	48.0 (39.0, 59.0)	0.15
MBP 6 h post surgery	65.0 (55.0, 75.0)	67.0 (59.0, 75.0)	53.0 (49.0, 66.0)	0.004
SBP 12 h post surgery	88.0 (78.0, 98.0)	89.0 (81.0, 100.0)	69.5 (62.0, 79.0)	< 0.001
DBP 12 h post surgery	51.0 (42.0, 58.0)	51.0 (44.0, 58.0)	51.0 (37.0, 58.0)	0.5
MBP 12 h post surgery	62.0 (55.0, 69.0)	63.0 (56.0, 69.0)	58.5 (44.0, 69.0)	0.049
SBP 24 h post surgery	91.0 (78.0, 104.0)	92.0 (79.0, 106.0)	81.5 (70.0, 92.0)	0.014
DBP 24 h post surgery	52.0 (44.0, 62.0)	52.0 (45.0, 62.0)	51.5 (40.0, 61.0)	0.6
MBP 24 h post surgery	65.0 (57.0, 77.0)	67.0 (57.0, 77.0)	63.5 (48.0, 71.0)	0.11

*CRT* capillary refill time, *SBP* systolic blood pressure, *DBP* diastolic blood pressure, *MBP* mean blood pressure

1. Median (Q1, Q3); *n* (%) 2. Wilcoxon rank sum test; Pearson’s Chi-squared test; Fisher’s exact test

Serum lactate was also an important predictor at all time points evaluated, with moderate but consistent associations, particularly at admission (HR 1.28, 95%CI 1.16–1.41, *p* < 0.001) and at 6 h (HR 1.32, 95%CI 1.18–1.46, *p* < 0.001). On the contrary, maintaining higher blood pressures, especially SBP, was associated with a lower risk of AKI. At 6 h postoperatively, each additional mmHg reduced the risk by 6% (HR 0.94, 95%CI 0.91–0.97, *p* < 0.001), a trend that persisted at 12 h (HR 0.96, 95%CI 0.94–0.98, *p* < 0.001). Kaplan–Meier curves describe AKI-free survival over time; associations with covariates were assessed using Cox models. Showing that the initial survival probability was close to 100%, with most declines occurring within the first 72 h after surgery. By day 8, AKI-free survival was approximately 85% (Supplementary Fig. 1).

**Fig. 2:**
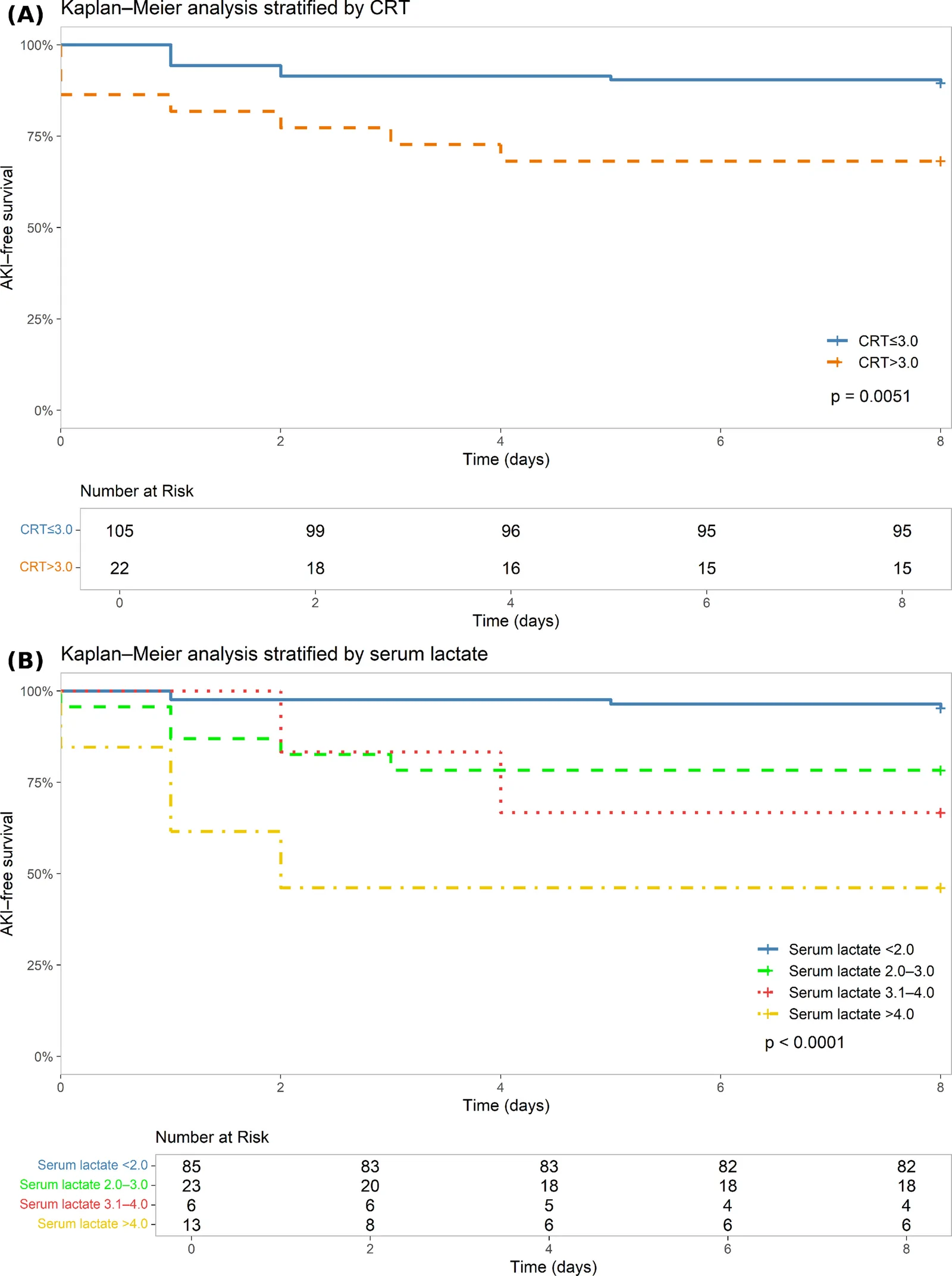
Kaplan–Meier survival curves stratified by capillary refill time (CRT) and serum lactate levels. **A** Survival curves stratified by CRT categories. **B** Survival curves stratified by serum lactate levels. The shaded areas represent 95% confidence intervals. *P*-values were calculated using the log-rank test. Numbers at risk are displayed below each curve at specific time intervals

After performing the stratified Kaplan–Meier analysis by CRT and serum lactate, significant differences in AKI-free survival were demonstrated when stratified by both perfusion markers (Fig. 2). The description of the categories is in Supplementary Table 2. The patients with prolonged CRT (> 3.0 s) showed markedly lower AKI-free survival compared to those with normal CRT (≤ 3.0 s) (*p* = 0.0051) (Fig. 2A). Similarly, serum lactate levels displayed a dose–response relationship with AKI development. Patients with lactate ≥ 4.0 mmol/L had substantially reduced AKI-free survival (approximately 50% by day 8), while those with lactate < 2.0 mmol/L maintained AKI-free survival above 90% (*p* < 0.0001) (Fig. 2B). The steepest decline in AKI-free survival occurred within the first 72 h postoperatively across all subgroups, consistent with the temporal patterns observed in the univariate analysis.

### Multivariate model

**Fig. 3:**
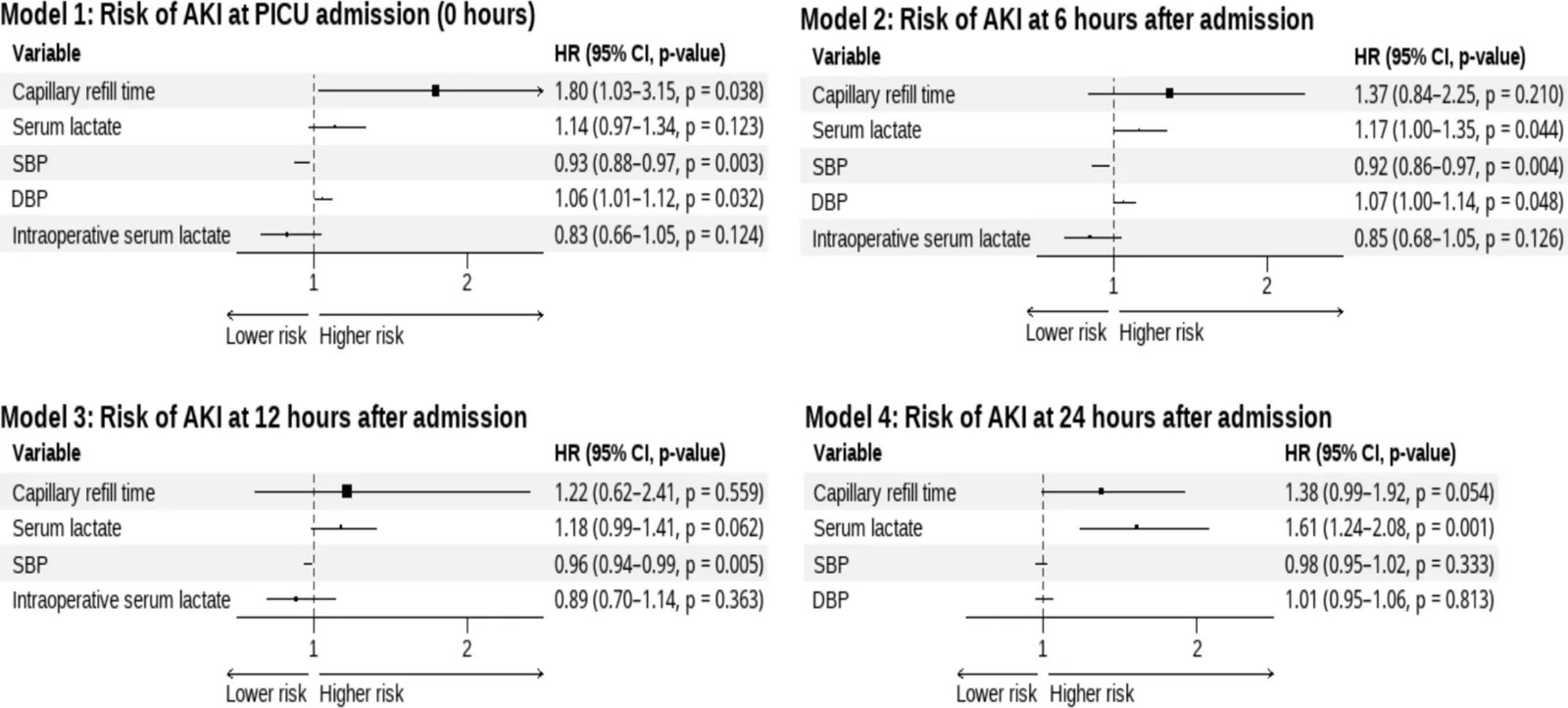
Forest plots of the hazard ratios (95%CI) for each of the multivariate models with the predictors of outcomes at admission, at 6, 12, and 24 h. The vertical dashed line represents an HR = 1.0. Variables with confidence intervals that do not cross the line are statistically significant (*p* < 0.05)

Cox proportional hazards models stratified by time point (cardiovascular ICU admission, 6, 12, and 24 h postoperatively) were implemented to evaluate the association between hemodynamic and metabolic variables with the development of AKI; all variables included in each model, as well as their results, are shown in Fig. 3. Furthermore, the proportional hazards assumption was verified using the Schoenfeld test across all models, and residual analysis identified no influential observations or evidence of inadequate model specification (Fig. 2). SBP demonstrated a significant protective effect during the first 12 h (admission: HR = 0.93, 95%CI 0.88–0.97, *p* < 0.05; 6 h: HR = 0.92, 95%CI 0.86–0.97, *p* < 0.05; 12 h: HR = 0.96, 95%CI 0.94–0.99, *p* < 0.05), which did not persist at 24 h (HR = 0.98, 95%CI 0.95–1.02, *p* > 0.05). Serum lactate showed a progressive increase in its risk association, reaching statistical significance at 24 h (admission: HR = 1.14, 95%CI 0.97–1.34; 6 h: HR = 1.17, 95%CI 1.00–1.35; 12 h: HR = 1.18, 95%CI 0.99–1.41; 24 h: HR = 1.61, 95%CI 1.24–2.08, *p* < 0.05) (Fig. 3).

Prolonged CRT constituted a significant risk factor at admission (HR = 1.80, 95%CI 1.03–3.15, *p* < 0.05), losing its significance at 24 h (HR = 1.38, 95%CI 0.99–1.92, *p* > 0.05). DBP was associated with increased risk in early assessments (admission: HR = 1.06, 95%CI 1.01–1.12; 6 h: HR = 1.07, 95%CI 1.00–1.14, *p* < 0.05), without maintaining significance at 24 h (HR = 1.01, 95%CI 0.95–1.06, *p* > 0.05) (Fig. 3).

## Discussion

AKI is a common complication following cardiovascular surgery in pediatric patients that is associated with increased morbidity, mortality, and prolonged hospital stay [6]. In our study, clinical characteristics associated with the appearance of AKI in critically ill patients undergoing cardiovascular surgery were evaluated. An incidence of AKI of 14.17% was found, which is within the range reported by various studies that have evaluated the incidence of this outcome in a similar population.

For example, Nagy et al. reported a 13.7% incidence of AKI in pediatric patients requiring cardiac surgery in the first 2 days postoperatively [18]. Also, Cardoso et al. reported an incidence of AKI of 12.3% in the first 24 h post-surgery [6].

In the literature, several factors associated with the development of AKI have been reported in the pediatric population requiring cardiovascular surgery. These findings are consistent with the concept that lactate may capture early metabolic stress and global hypoperfusion relevant to AKI pathophysiology, particularly given the delayed nature of creatinine- and urine output-based AKI criteria, as highlighted in recent work on lactate metabolism as an early biomarker approach [19, 35]. Locally, Restrepo de Rovetto et al. reported that in a cohort of 485 pediatric patients undergoing cardiovascular surgery between 2012 and 2014, RACHS score greater than or equal to 3 points, ECMO time, and invasive mechanical ventilation time were associated with AKI [5].

Also, in a systematic review and meta-analysis carried out in 2021 by the International Association of Pediatric Nephrology, sepsis, RACHS greater than or equal to 3 points and longer duration of ECMO were evidenced as major risk factors for the incidence of AKI [20]. In our cohort, perfusion-related variables remained relevant after adjustment. In the multivariate Cox models, systolic blood pressure showed a protective association during the first 12 postoperative hours, while serum lactate became significantly associated with AKI at 24 h (HR 1.61, 95%CI 1.24–2.08). Prolonged CRT was independently associated with AKI at ICU admission (HR 1.80, 95%CI 1.03–3.15).

On the contrary, exposure to nephrotoxic drugs has also been reported as a risk factor for the presence of AKI after cardiovascular surgery [21]. The mechanism by which nephrotoxic drugs produce AKI is widely described in the literature and includes vasoconstriction or hemodynamic alterations within the glomerulus, direct tubular damage, interstitial nephritis, crystal formation and deposition, glomerular injury including podocytopathy, and osmotic nephrosis [22, 23].

In a study conducted in Buenos Aires, Graziani et al. reported an incidence of AKI of 35%, of which 57% of patients had AKI on the first postoperative day. In these patients, the factors associated with AKI were exposure to vancomycin (*p* = 0.012) and thiazide diuretics (*p* = 0.015) [14]. In relation to exposure to nephrotoxic drugs, our results did not reveal significant patterns in pediatric patients undergoing cardiovascular surgery since exposure to nephrotoxic drugs was more frequent in patients who did not develop AKI.

Mortality in our study was 44.4% in patients with AKI, evidencing a strong relationship between the development of this complication and the risk of in-hospital death. This is consistent with what has been reported in the scientific literature, which indicates that the occurrence of AKI was a risk factor for death (OR 5.82; 95%CI 2.24–15.10; *p* < 0.001) [24]. Mortality has been a shocking outcome in studies of AKI and cardiovascular surgery. In a prospective study from 2009 to 2017 by Hames et al., they found a mortality of 75%, with the prolonged time between the presentation of AKI and the initiation of kidney replacement therapy being the most important associated factor of mortality [25].

The mortality of our cohort was high but expected compared to that reported in other studies carried out in southwestern Colombia. In our hospital, in studies conducted in the period prior to the use of pRIFLE (1996–2006), Restrepo et al. reported a high mortality in patients who underwent cardiovascular surgery and presented AKI: 70% between 1996 and 1999, 75% between 2000 and 2002, and 50% between 2003 and 2006 [26]. After the introduction of pRIFLE (2009–2012), Restrepo et al. reported a mortality of 30% in patients who developed AKI after cardiovascular surgery [27], and Restrepo de Rovetto et al., using AKIN retrospectively, reported a mortality of 44.9% in patients with AKI after cardiovascular surgery [5].

We observed a 14.17% incidence of AKI, consistent with previous studies such as that of Aoun et al., who documented an incidence of 9.3% when evaluating patients with AKI after cardiovascular surgery in a high-complexity hospital in a low- to middle-income country like ours [28]. In addition, Aoun et al. reported that patients with cyanotic congenital heart disease show a higher prevalence of AKI (78% vs. 44%) compared to patients who have non-cyanotic congenital heart disease [28].

Secondary outcomes such as duration of mechanical ventilation, ICU and hospital length of stay, and need for postoperative ECMO were significantly worse among patients who developed AKI. These findings underscore the broad clinical impact of AKI beyond renal function, as it reflects a systemic deterioration requiring prolonged intensive care and increased resource use. Additionally, the 28-day mortality rate in patients with AKI was markedly higher (44.4%) compared to those without (7.3%), reinforcing the association between postoperative AKI and poor outcomes in critically ill pediatric populations.

Analysis of clinical features revealed that both capillary filling and lactate levels correlate closely with the development of AKI in the postoperative period. Patients who developed AKI had significantly higher serum lactate levels at admission, 6, 12, and 24 h after the surgical procedure, and a longer CRT compared to those who did not experience this complication. Maarslet et al. conducted a retrospective observational study in Denmark in 206 children who underwent cardiovascular surgery (2006–2007) and reported that 8% had serum lactate levels greater than 4.5 mmol/l and that these levels showed an odds ratio (95%CI) of 8.4 (1.5–46.1) for mortality [29].

More recently and similarly, Aoun et al., reported a statistically significant difference in serum lactate levels between patients with and without AKI (6.8 ± 6.9 vs. 2.85 ± 1.55 mmol/L; *p* = 0.03), suggesting the potential usefulness of this biomarker as an early indicator of renal dysfunction [28].

Our findings align with similar prior studies; a Chilean study found that intraoperative hyperlactatemia during cardiopulmonary bypass (lactate > 6 mmol/L) has been associated with postoperative acute renal failure, suggesting that the association may begin intraoperatively and persist into the early ICU course [30, 34].

In a large multicenter randomized clinical trial in early septic shock (ANDROMEDA-SHOCK-2), a personalized hemodynamic resuscitation strategy targeting CRT normalization was superior to usual care for a hierarchical composite outcome at 28 days. Although this evidence comes from adults, it supports CRT as a clinically meaningful, actionable perfusion marker. In the postoperative period after pediatric cardiovascular surgery where transient low cardiac output, systemic inflammation, and hypoperfusion are common, early perfusion markers such as CRT, together with serum lactate as a marker of global tissue hypoxia, may help identify children at higher risk of developing AKI [31, 36].

Lactate and CRT should be interpreted as bedside markers of global and peripheral perfusion adequacy, rather than direct surrogates of renal blood flow. Persistent abnormalities in lactate and CRT likely identify patients experiencing low cardiac output syndrome, shock physiology, or impaired oxygen delivery, all of which can contribute to kidney hypoxia and susceptibility to cardiac surgery-associated AKI through hemodynamic and inflammatory pathways. Follow-up on the improvement of these parameters after patient recovery is an important aspect that would better be addressed in future research to expand the findings of our study.

These statistically and clinically significant differences allow the identification of early, frequently used, and easily accessible markers and factors associated with AKI, which could facilitate monitoring and prevention strategies in patients undergoing pediatric cardiovascular surgery.

## Limitations

This study has several limitations. First, its single-center design may limit the generalizability of the findings to other settings. Second, although multivariable models were applied, residual confounding due to unmeasured variables cannot be excluded. Third, the sample size was relatively small, which may reduce statistical power and increase the risk of type II error. Fourth, interactions between predictors were not explored, and follow-up was limited, preventing assessment of long-term outcomes. Future multicenter studies with larger cohorts and extended follow-up are needed to validate these findings and refine risk prediction.

## Conclusions

This study describes the incidence and factors associated with AKI in pediatric patients undergoing cardiovascular surgery in the ICU of a high-complexity center in southwestern Colombia. The incidence of AKI was similar to that reported in the scientific evidence available in our setting. Our findings highlight the need to implement close surveillance in these patients, both during the surgical and postoperative period, and to use markers such as serum lactate and capillary filling to improve the early detection of tissue injury, including AKI. Creating a protective environment for the kidney during hospital stay and initiating early treatment are critical strategies to prevent the progression of AKI and optimize renal recovery before hospital discharge.

## Supplementary Information

Below is the link to the electronic supplementary material.

ESM 1 DOCX (141 KB)

Graphical abstract (PPTX 188 KB) 

## Data Availability

Deidentified data and analysis code are available from the corresponding author on reasonable request.

## Abbreviations

AKI: Acute kidney injury
CS: Cardiovascular surgery
CRT: Capillary refill time
ECMO: Extracorporeal membrane oxygenation
ICU: Intensive care unit
KDIGO: Kidney Disease: Improving Global Outcomes
HR: Hazard ratio
PRISM: Pediatric Risk of Mortality
RACHS1: Risk Adjustment for Congenital Heart Surgery
GFR: Glomerular filtration rate

## References

[1] Susantitaphong P, Cruz DN, Cerda J, Abulfaraj M, Alqahtani F, Koulouridis I et al (2013) World incidence of AKI: a meta-analysis. Clin J Am Soc Nephrol 8:1482–1493 10.2215/CJN.00710113 PMC3805065 23744003

[2] Mehta RL, Cerdá J, Burdmann EA, Tonelli M, García-García G, Jha V et al (2015) International Society of Nephrology’s 0by25 initiative for acute kidney injury (zero preventable deaths by 2025): a human rights case for nephrology. Lancet 385:2616–2643 10.1016/S0140-6736(15)60126-X 25777661

[3] ​Restrepo JM, Mondragon MV, Forero-Delgadillo JM, Lasso RE, Zemanate E, Bravo Y et al (2020) Acute renal failure in children. Multicenter prospective cohort study in medium-complexity intensive care units from the Colombian southeast. PLoS One 15:e0235976 10.1371/journal.pone.0235976 PMC7446789 32833971

[4] Van den Eynde J, Delpire B, Jacquemyn X, Pardi I, Rotbi H, Gewillig M et al (2022) Risk factors for acute kidney injury after pediatric cardiac surgery: a meta-analysis. Pediatr Nephrol 37:509–519 10.1007/s00467-021-05297-0 34595570

[5] Restrepo-De Rovetto C, Llanos C, Silva G, Martínez A, Torres M, Muñoz C et al (2022) Incidence and risk factors for acute kidney injury associated to surgery for congenital heart disease. Revista Colombiana de Cardiologia 29:70–76

[6] Cardoso B, Laranjo S, Gomes I, Freitas I, Trigo C, Fragata I et al (2016) Insuficiência renal aguda no contexto de cirurgia cardíaca pediátrica: fatores de risco e prognóstico. Proposta de um modelo preditivo. Rev Port Cardiol 35:99–104 10.1016/j.repc.2015.06.006 26831910

[7] Sharma A, Chakraborty R, Sharma K, Sethi SK, Raina R (2020) Development of acute kidney injury following pediatric cardiac surgery. Kidney Res Clin Pract 39:259–268 10.23876/j.krcp.20.053 PMC7530361 32773391

[8] Kumar Sethi S, Kumar M, Sharma R, Bazaz S, Kher V (2015) Acute kidney injury in children after cardiopulmonary bypass: risk factors and outcome. Indian Pediatr 52:223–226 10.1007/s13312-015-0611-4 25848999

[9] Basaran M, Sever K, Kafali E, Ugurlucan M, Sayin OA, Tansel T et al (2006) Serum lactate level has prognostic significance after pediatric cardiac surgery. J Cardiothorac Vasc Anesth 20:43–47 10.1053/j.jvca.2004.10.010 16458212

[10] Pettit KA, Melink KF, Alten JA, Goldstein SL, Ollberding N, SooHoo M et al (2024) Biomarker-based acute kidney injury sub-phenotypes refine risk assessment in children undergoing cardiac surgery. Pediatr Nephrol 40:523–531 10.1007/s00467-024-06541-z PMC12358096 39331076

[11] Fleming S, Gill P, Jones C, Taylor JA, Van den Bruel A, Heneghan C, Roberts N, Thompson M (2015) The diagnostic value of capillary refill time for detecting serious illness in children: a systematic review and meta-analysis. PLoS One 10:e0138155. 10.1371/journal.pone.0138155 PMC4573516 26375953

[12] Hernandez G, Pedreros C, Veas E, Bruhn A, Romero C, Rovegno M, Neira R, Bravo S, Castro R, Kattan E, Ince C (2012) Evolution of peripheral vs metabolic perfusion parameters during septic shock resuscitation. A clinical-physiologic study. J Crit Care 27:283–288. 10.1016/j.jcrc.2011.05.024 21798706

[13] Raia L, Gabarre P, Bonny V, Urbina T, Missri L, Boelle PY, Baudel JL, Guidet B, Maury E, Joffre J, Ait-Oufella H (2022) Kinetics of capillary refill time after fluid challenge. Ann Intensive Care 12:74. 10.1186/s13613-022-01049-x PMC9375797 35962860

[14] Paula Graziani M, Moser M, Martín Bozzola C, Galvez HM, Garrido JI, Guido Álvarez P et al (2019) Acute kidney injury in children after cardiac surgery: risk factors and outcomes. A retrospective cohort study. Arch Argent Pediatr 117:E557–567 10.5546/aap.2019.eng.e557 31758882

[15] Cruz G, Pedroza Gómez S, Arango A, Guevara PA, González C, Aguirre J et al (2023) Capillary Refill time and serum lactate as predictors of mortality and postoperative extracorporeal membrane oxygenation requirement in congenital heart surgery. Children (Basel) 10:875 10.3390/children10050875 PMC10217530 37238423

[16] Schwartz GJ, Muñoz A, Schneider MF, Mak RH, Kaskel F, Warady BA, Furth SL (2009) New equations to estimate GFR in children with CKD. J Am Soc Nephrol 20:629–637. 10.1681/ASN.2008030287 PMC2653687 19158356

[17] Li S, Krawczeski CD, Zappitelli M, Devarajan P, Thiessen-Philbrook H, Coca SG et al (2011) Incidence, risk factors, and outcomes of acute kidney injury after pediatric cardiac surgery: a prospective multicenter study. Crit Care Med 39:1493–1499 10.1097/CCM.0b013e31821201d3 PMC3286600 21336114

[18] Nagy M, Onder AM, Rosen D, Mullett C, Morca A, Baloglu O (2024) Predicting pediatric cardiac surgery-associated acute kidney injury using machine learning. Pediatr Nephrol 39:1263–1270 10.1007/s00467-023-06197-1 37934270

[19] Shen F, Xu Y, Jiang X, Yu L, Ge HH, Wang X, Sun YQ (2025) Predicting outcomes in pediatric patients with acute kidney injury: a retrospective single-center cohort study using machine learning models. BMC Med Inform Decis Mak 25:371. 10.1186/s12911-025-03211-4 PMC12512778 41074021

[20] Mendelson J (2018) Emergency department management of pediatric shock. Emerg Med Clin North Am 36:427–440 10.1016/j.emc.2017.12.010 29622332

[21] Lu H, Thurnherr E, Meaney CJ, Fusco NM (2021) Incidence and risk factors for acute kidney injury in hospitalized children receiving piperacillin-tazobactam. J Pediatr Pharmacol Ther 26:597–602 10.5863/1551-6776-26.6.597 PMC8372863 34421409

[22] Kwiatkowska E, Domański L, Dziedziejko V, Kajdy A, Stefańska K, Kwiatkowski S (2021) The mechanism of drug nephrotoxicity and the methods for preventing kidney damage. Int J Mol Sci 22:6109 10.3390/ijms22116109 PMC8201165 34204029

[23] Uber AM, Sutherland SM (2020) Nephrotoxins and nephrotoxic acute kidney injury. Pediatr Nephrol 35:1825–1833. 10.1007/s00467-019-04397-2 31646405

[24] Uber AM, Sutherland SM (2020) Acute kidney injury in hospitalized children: consequences and outcomes. Pediatr Nephrol 35:213–220 10.1007/s00467-018-4128-7 PMC7223774 30386936

[25] Hames DL, Ferguson MA, Kaza AK, Rajagopal S, Thiagarajan RR, Teele SA et al (2019) Renal replacement therapy in the pediatric cardiac intensive care unit. J Thorac Cardiovasc Surg 158:1446–1455 10.1016/j.jtcvs.2019.06.061 31395365

[26] Restrepo R JM, Villareal N, Leyes D, Botero Á, Daza V, Manzi E et al (2015) Prevalencia y caracterización de la población con insuficiencia renal aguda en la UCIP de la Fundación Valle del Lili enero. Arch Latin Nefr Ped 15

[27] Restrepo JM, Castillo G, Cañas A, Duque M del P, Ramirez O (2012) Detección y prevención temprana de la falla renal aguda en pacientes que ingresaron a la unidad de cuidado intensivo pediátrico y cardiovascular de la Fundación Clínica Valle del Lili. Acta Colombiana de Cuidado Intensivo 12:220–225

[28] Aoun B, Daher GA, Daou KN, Sanjad S, Tamim H, El Rassi I et al (2021) Acute kidney injury post-cardiac surgery in infants and children: a single-center experience in a developing country. Front Pediatr 9:637463 10.3389/fped.2021.637463 PMC8350128 34381742

[29] Maarslet L, Møller MB, Dall R, Hjortholm K, Ravn H (2012) Lactate levels predict mortality and need for peritoneal dialysis in children undergoing congenital heart surgery. Acta Anaesthesiol Scand 56:459–464 10.1111/j.1399-6576.2011.02588.x 22150620

[30] Reyes-Flandes EN, Herrera-Landero A, Bobadilla-González P, Núñez-Enríquez JC (2017) Factores de riesgo asociados a insuficiencia renal aguda postoperatoria en pacientes pediátricos intervenidos de cirugía cardiaca que requirieron de circulación extracorpórea [Risk factors associated with postoperative acute renal failure in pediatric patients undergoing cardiopulmonary bypass surgery]. Rev Chil Pediatr 88:209–215. 10.1016/j.rchipe.2016.08.004 28542655

[31] The ANDROMEDA-SHOCK-2 Investigators for the ANDROMEDA Research Network, Spanish Society of Anesthesiology, Reanimation and Pain Therapy (SEDAR), and Latin American Intensive Care Network (LIVEN) (2025) Personalized hemodynamic resuscitation targeting capillary refill time in early septic shock: the ANDROMEDA-SHOCK-2 randomized clinical trial. JAMA 334:1988–1999. 10.1001/jama.2025.20402 PMC12573117 41159835

[32] ​Pantoja-Gómez OC, Realpe S, Cabra-Bautista G, Restrepo JM, Prado OL, Velasco AM et al (2022) Clinical course of neonatal acute kidney injury: multi-center prospective cohort study. BMC Pediatr 22:136 10.1186/s12887-022-03200-w PMC8920800 35287608

[33] ​Macedo E, Cerdá J, Hingorani S, Hou J, Bagga A, Burdmann EA et al (2018) Recognition and management of acute kidney injury in children: the ISN 0by25 Global Snapshot study. PLoS One 13:e0196586 10.1371/journal.pone.0196586 PMC5929512 29715307

[34] ​Sutherland SM, Byrnes JJ, Kothari M, Longhurst CA, Dutta S, Garcia P et al (2015) AKI in hospitalized children: comparing the pRIFLE, AKIN, and KDIGO definitions. Clin J Am Soc Nephrol 10:554–561 10.2215/CJN.01900214 PMC4386245 25649155

[35] ​Beken S, Akbulut BB, Albayrak E, Güner B, Ünlü Y, Temur B et al (2021) Evaluation of neonatal acute kidney injury after critical congenital heart disease surgery. Pediatr Nephrol 36:1923–1929 10.1007/s00467-020-04890-z 33492453

[36] ​Alten JA, Cooper DS, Blinder JJ, Selewski DT, Tabbutt S, Sasaki J et al (2021) Epidemiology of acute kidney injury after neonatal cardiac surgery: a report from the multicenter neonatal and pediatric heart and renal outcomes network. Crit Care Med 49:E941–951 10.1097/CCM.0000000000005165 34166288

[37] Reddy RK, Menon S, Selewski DT (2024) Putting the injury back into AKI: understanding AKI phenotypes and improving risk assessment in cardiac surgery–associated AKI. Pediatr Nephrol 40:887–890 10.1007/s00467-024-06617-w 39656275

