# Supplementary material for: Serum lactate and capillary refill time as predictors of acute kidney injury after pediatric cardiovascular surgery: a prospective cohort study
Source: Pediatr Nephrol. 2026 Jul 23;41(12):4677–88. doi: 10.1007/s00467-026-07303-9 (PMC13672414; doi:10.1007/s00467-026-07303-9)
Supplement: Supplementary file 1 [file 467_2026_7303_MOESM1_ESM.docx]

| **Supplementary Table 1. Characteristics of the surgical intervention performed** | **Patients without acute kidney injury** | **Patients with acute kidney injury** |
| --- | --- | --- |
|  | **n=109** | **n=18** |
| Type of procedure |  |  |
| Elective | 64 (58.7) | 3 (16.7) |
| Urgent/emergency | 45 (41.3) | 15 (83.3) |
| Closing of Ventricular Septal Defect | 19 (17.4) | 1 (5.6) |
| Atrial Septal Defect closure | 13 (11.9) | - |
| Norwood Surgery | 4 (3.7) | 2 (11.1) |
| Jatene procedure | 3 (2.8) | 3 (16.7) |
| Systemic-to-pulmonary shunt | 4 (3.7) | 1 (5.6) |
| Pulmonary homograft | 4 (3.7) | - |
| Correction of abnormal venous connection | 3 (2.8) | - |
| Glenn procedure | 10 (9.2) | 1 (5.6) |
| Aortic repair | 7 (6.4) | 1 (5.6) |
| Subaortic membrane resection | 5 (4.6) | - |
| Truncus arteriosus repair | 2 (1.8) | - |
| Aortic valve surgery | 3 (2.8) | 1 (5.6) |
| Mitral valve surgery | 2 (1.8) | 1 (5.6) |
| Pulmonary valve surgery | 6 (5.5) | 1 (5.6) |
| Subclavian repair | 1 (0.9) | - |
| Tricuspid valve surgery | - | - |
| Atrioseptostomy | 1 (0.9) | - |
| Atrioventricular canal defect closure | 5 (4.6) | 1 (5.6) |
| Closure of Ventricular Septal Defect + Atrial Septal Defect | 2 (1.8) | - |
| Ventricular Septal Defect + Atrial Septal Defect + plasty closure | 1 (0.9) | - |
| Atrial Septal Defect closure + lung plasty | 1 (0.9) | - |
| Lung Cerclage Removal | 3 (2.8) | - |
| Fontan procedure | 3 (2.8) | 1 (5.6) |
| Tetralogy of Fallot Correction Surgery | 3 (2.8) | - |
| Anomalous Left Coronary Artery from the Pulmonary Artery Anomaly Correction | - | 1 (5.6) |
| Aortopulmonary window repair | 1 (0.9) | - |
| Intracardiac foreign body removal | - | 1 (5.6) |
| Norwood surgery + atrioseptostomy | - | - |
| Reconstruction of the aortic arch + pulmonary artery buckle | 1 (0.9) | - |
| Resection of fungal endocardial seedings in the carotid sinus | 1 (0.9) | - |
| Pulmonary artery cerclage | 1 (0.9) | - |
| Damus-Kaye-Stansel Surgery | - | 1 (5.6) |
| Right Ventricle outflow tract enlargement + infundibular resection + transannular patch + Ventricular Septal Defect closure + collateral ligation | - | 1 (5.6) |

Supplementary Figure 1.

**Supplementary Figure 1. Kaplan–Meier curve for AKI-free survival during the first 8 postoperative days.** The curve illustrates the cumulative probability of remaining free from acute kidney injury (AKI) over time. The initial survival probability was close to 100%, with most declines occurring within the first 72 hours after surgery. By day 8, AKI-free survival was approximately 85%. The curve represents the entire cohort.

**Supplementary Table 2. Categories developed for CRT and serum lactate for Kaplan–Meiner analysis**

| **Variable** |  | **Acute kidney injury** | | **p-value***^2^* |
| --- | --- | --- | --- | --- |
|  | **Overall** | **0. No** | **1. Yes** |  |
|  | N = 127*^1^* | N = 109*^1^* | N = 18*^1^* |  |
| **Median CRT** | 2.5 (2.0, 3.0) | 2.5 (2.0, 2.8) | 3.0 (2.8, 3.5) | <0.001 |
| **Capillary refill time** |  |  |  | 0.016 |
| 0. CRT≤3.0 | 105 (82.7%) | 94 (86.2%) | 11 (61.1%) |  |
| 1. CRT>3.0 | 22 (17.3%) | 15 (13.8%) | 7 (38.9%) |  |
| **Median serum lactate** | 1.5 (1.2, 2.2) | 1.4 (1.2, 2.0) | 2.8 (2.1, 6.8) | <0.001 |
| **Serum lactate** |  |  |  | <0.001 |
| 0. <2.0 | 85 (66.9%) | 81 (74.3%) | 4 (22.2%) |  |
| 1. 2.0–3.0 | 23 (18.1%) | 18 (16.5%) | 5 (27.8%) |  |
| 2. 3.1–4.0 | 6 (4.7%) | 4 (3.7%) | 2 (11.1%) |  |
| 3. >4.0 | 13 (10.2%) | 6 (5.5%) | 7 (38.9%) |  |
| *^1^* Median (Q1, Q3); n (%) | | | | |
| *^2^* Wilcoxon rank sum test; Fisher’s exact test | | | | |
